# A Literature Review of Host Feeding Patterns of Invasive *Aedes* Mosquitoes in Europe

**DOI:** 10.3390/insects11120848

**Published:** 2020-11-29

**Authors:** Sonia Cebrián-Camisón, Josué Martínez-de la Puente, Jordi Figuerola

**Affiliations:** 1 Estación Biológica de Doñana, Departamento de Ecología de Humedales, Av. Américo Vespucio 26, 41092 Sevilla, Spain; jordi@ebd.csic.es; 2 Departamento de Parasitología, Facultad de Farmacia, Campus Universitario de Cartuja, Universidad de Granada, 18071 Granada, Spain; 3 Centro de Investigación Biomédica en Red de Epidemiología y Salud Pública (CIBERESP), 28029 Madrid, Spain

**Keywords:** alien species, Asian tiger mosquito, dengue, feeding pattern, feeding behavior, hosts, vectors, yellow fever, zika

## Abstract

**Simple Summary:**

Invasive mosquito species alter the local epidemiology of many pathogens in the invaded areas, including locality circulating pathogens and imported ones. Four invasive species of the genus *Aedes* are established in Europe, potentially affecting the transmission of vector-borne diseases in the area. These species include *Aedes aegypti*, *Aedes albopictus*, *Aedes japonicus* and *Aedes koreicus*. Here, we extensively review the blood feeding patterns of these invasive *Aedes* mosquitoes which constitute a key parameter affecting the contact rates between infected and susceptible hosts, thus playing a central role in epidemiology of mosquito-borne pathogens. Our results show that these mosquito species feed on different vertebrate groups, especially on mammals. Humans are common hosts of these species, representing 36% and 93% of the blood meals identified for *Aedes japonicus* and *Aedes aegypti*, respectively. Birds and, even, ectotherms have been recorded as potential hosts of these *Aedes* invasive mosquitoes. Given their competence for the transmission of emerging arboviruses such as dengue or Chikungunya viruses and their rates of feeding in humans, *Aedes* invasive species may have an important impact in the transmission of these pathogens in urban and periurban areas. Finally, we identify the knowledge gaps on the blood feeding patterns of these species and propose directions for future research.

**Abstract:**

*Aedes* invasive mosquitoes (AIMs) play a key role as vectors of several pathogens of public health relevance. Four species have been established in Europe, including *Aedes aegypti*, *Aedes* *albopictus*, *Aedes japonicus* and *Aedes koreicus*. In addition, *Aedes atropalpus* has been repeatedly recorded although it has not yet been established. In spite of their importance in the transmission of endemic (e.g., heartworms) and imported pathogens (e.g., dengue virus), basic information of parameters affecting their vectorial capacity is poorly investigated. The aim of this study is to review the blood feeding patterns of these invasive mosquito species in Europe, summarizing available information from their native and introduced distribution ranges. The feeding patterns of mosquitoes constitute a key parameter affecting the contact rates between infected and susceptible hosts, thus playing a central role in the epidemiology of mosquito-borne pathogens. Our results highlight that these mosquito species feed on the blood of different vertebrate groups from ectotherms to birds and mammals. However, humans represent the most important source of blood for these species, accounting for 36% and 93% of hosts identified for *Ae. japonicus* and *Ae. aegypti*, respectively. In spite of that, limited information has been obtained for some particular species, such as *Ae*. *koreicus*, or it is restricted to a few particular areas. Given the high vector competence of the four AIM species for the transmission of different emerging arboviruses such as dengue, Chikungunya, Zika or Yellow fever viruses and their high feeding rates on humans, these AIM species may have an important impact on the vectorial capacity for such pathogens on urban and periurban areas. Finally, we propose directions for future research lines based on identified knowledge gaps.

## 1. Introduction

Mosquitoes (family *Culicidae*) are almost ubiquitous, being absent only from some remote areas such as Antarctica. Mosquitoes are a nuisance for humans because of their bites but they also transmit many pathogens to humans and other animals [1,2]. Among vector-borne diseases, mosquito-borne pathogens are particularly relevant causing malaria, dengue fever, Yellow fever, Japanese encephalitis and lymphatic filariasis, among many other diseases [3,4]. For example, malaria alone is responsible for significant rates of morbidity with approximately 405,000 fatalities annually [5]. Although mosquitoes are particularly abundant in the humid tropics and subtropics, they also represent a public health concern in temperate areas [6,7]. Nowadays, the public and scientific concern on mosquito-borne diseases is increasing as new diseases emerge and others resurge or expand to new geographic areas [8]. This expansion of vector borne diseases is often fuelled by processes of invasion by mosquito species with the capacity to transmit pathogens with large relevance for public health and well adapted to proliferate in urban environments [9,10,11]. For example, the Asian tiger mosquito *Aedes albopictus* is a well-known vector of pathogens including dengue, Zika and Chikungunya viruses [12,13] and has been involved in dengue outbreaks in France in 2010, Spain in 2018 and in Italy in 2020 [14,15,16], Chikungunya outbreaks in Italy [17] and the local transmission of Zika virus in France [18]. *Aedes albopictus* has also been involved in the transmission of autochthonous locally circulating pathogens such as the nematode *Dirofilaria immitis* in Italy [19]. In addition, mosquito invasions and the pathogens they can transmit may also have important negative impact on wildlife populations. This is the case of the introduction of *Culex pipiens* in the Hawaii archipelago that has allowed the local transmission of the avian malaria parasite *Plasmodium relictum* among immunologically naïve endemic avian species [20]. This parasite significantly contributed to the decline of native bird populations [21].

The aim of this study is to review the published information on the blood feeding patterns of invasive mosquitoes in Europe. In particular, we focus on species of the genus *Aedes*, which are vectors of both introduced and native pathogens of public health relevance [9,10,22]. The blood feeding patterns of mosquitoes is a key component in the estimation of their vectorial capacity as its study allows the identification of the potential vertebrate hosts of mosquitoes, estimate contact rates and represent an essential component in epidemiological studies of mosquito-borne pathogens [23,24]. In fact, human-biting rate is an important parameter for the estimation of the basic reproduction rate (R_0_) of vector borne pathogens like arboviruses, i.e., arthropod-borne viruses. R_0_ is defined as the average number of new cases expected from an infected individual placed in a population of susceptible hosts [25]. When referring to mosquito-borne diseases affecting humans, other important variables affecting R_0_ are vector longevity, pathogen development time and vector competence, i.e., the ability of mosquitoes to get infected following an infected blood meal and being able to transmit the pathogen during subsequent bites [26]. *Aedes* species are becoming a global concern due to their expansion throughout the globe, in particular in the case of *Aedes albopictus* and *Aedes aegypti,* which have already spread through the tropics, eastern Asia, Europe and North America [27].

## 2. *Aedes* Invasive Mosquitoes in Europe

**Figure 1:**
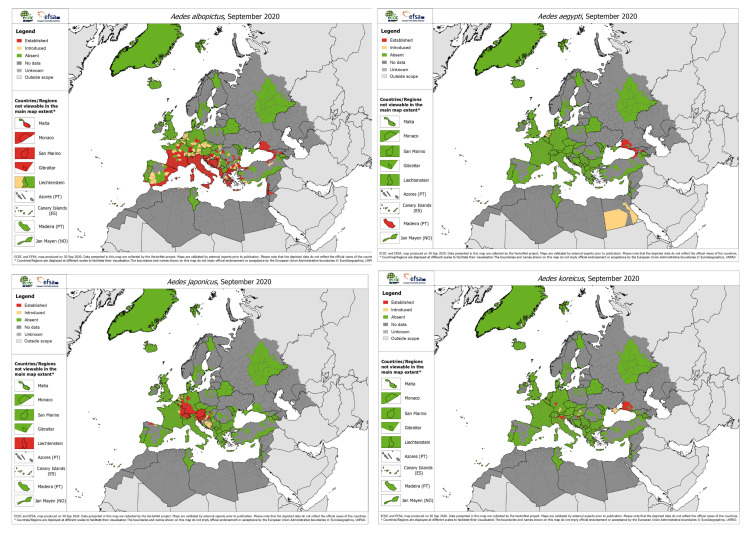
Current known distribution of *Aedes* invasive species in Europe (September 2020; accessed on 19 November 2020). The maps show the current European distribution of *Ae. albopictus* (upper-left panel), *Ae. aegypti* (upper-right panel), *Ae. japonicus* (lower-left panel) and *Ae. koreicus* (lower-left panel). European Centre for Disease Prevention and Control and European Food Safety Authority. Mosquito maps (internet). Stockholm: ECDC; 2020. Available from: https://ecdc.europa.eu/en/disease-vectors/surveillance-and-disease-data/mosquito-maps.

In recent decades, there have been continuous introduction events of exotic mosquitoes into Europe, facilitated by the global movements of people and goods [28,29,30]. Nowadays, there are four *Aedes* invasive mosquito (AIM) species with known established populations in Europe, namely, *Ae. albopictus, Ae. aegypti, Aedes japonicus* and *Aedes koreicus* [31,32,33,34] (Figure 1). In addition, *Aedes atropalpus* has been detected in several occasions in different European countries such as France and the Netherlands although it has not been established yet [35].

*Aedes albopictus* is native to Southeast Asia but has spread its distribution to areas around the globe in the last 40 years. In the 19th century, *Ae. albopictus* colonized some islands of the Indian and the Pacific Ocean around its native range with the aid of human activities. This species was first detected in Europe (Albania) in 1979 and during the decade of the 1980s, new populations were established in North and South America and Africa [36,37,38,39]. This rapid spread has been possible due to the international trade, primarily of used tires [40,41] but also in other kind of shipments such as lucky bamboo, *Dracaena sanderiana* [42,43]. Further spread within countries may be facilitated by passive transport in vehicles [44]. Nowadays, *Ae. albopictus* is widely spread and established in more than 15 European countries [31] including Spain [45], France [46], Italy [47], Malta [48,49], Greece [50] and Montenegro [51].

*Aedes aegypti*, the yellow fever mosquito, was thought to be native to Africa, but now there is genetic evidence of its origin from Madagascar [52]. Nowadays, it is one of the most globally widespread mosquito species [9]. Its disseminations likely started in the 16th century, linked to the slave trade between Africa and the Americas [53]. In the early 20th century, this species occurred in European countries of the Mediterranean basin such as Spain, Greece, France, Italy, among others, but was eradicated from the area after 1950s, probably due to the malaria winter control campaigns [54,55]. Nowadays, *Ae. aegypti* is present in the Madeira islands [56] and in more eastern countries like Georgia, northeastern Turkey [57], and southern Russia [32].

*Aedes japonicus* was originally distributed in southern China, Korea, Japan, Taiwan and southern Russia [58]. Of the four known subspecies of *Ae. japonicus*, only *Aedes j. japonicus,* native to Japan, Korea, and Russian Primorsky Krai region, has become invasive [58]. *Aedes japonicus* was found established outside its native range for the first time in the United States [59] while the first report in Europe was in France in 2000 [60]. Since then, established populations of this mosquito species have also been detected in Belgium [61], Germany, Switzerland [62], Austria, Slovenia, Croatia [63], the Netherlands [64], Italy, Hungary [33] and Luxembourg [58]. More recently, thanks to a citizen science platform, *Ae. japonicus* has been also recorded in some regions of North Spain, where it is currently established [65].

*Aedes koreicus* is native to Asia, being present in South Korea, Japan, China, and eastern Russia [66]. This species was first detected outside its range in Belgium in 2008, where it is currently established [67]. Since then, the species has been recorded in Italy [68], Slovenia [69], Germany [70], European Russia [71], Hungary [72], and Switzerland [73]. The species is nowadays established in all the mentioned countries except Slovenia and Switzerland [34].

Finally, the American rock pool mosquito *Ae. atropalpus*, native from eastern North America, expanded its distribution in America due to the utilization of tires as breeding sites and the commerce of used tires through the continent [9]. In the 1990s, this species was reported in northern Italy in a used tires wholesale that imported tires from North America [28]. However, the rapid implementation of control treatments avoided the establishment of the population [47]. *Aedes atropalpus* was subsequently reported in 2003 and 2005 in France, and in the Netherlands in 2009, but in all the cases the populations were eradicated [9,74]. Nowadays, there are not known established populations of *Ae. atropalpus* in Europe, although it is introduced in the south of the Netherlands [35].

## 3. Methods Used for the Identification of Vertebrate Hosts of Invasive *Aedes* Mosquitoes

Mosquito species differ in their feeding preferences, which determine their contact rates with both pathogens and vertebrate hosts [75,76]. Due to the differential vector competence of mosquitoes and host susceptibility for pathogens, knowledge on the feeding patterns of mosquitoes provides valuable information to identify the key vectors of pathogens, its main reservoirs and also to assess the risk of transmission to humans and other target species. In the case of AIMs, studying their blood-feeding preferences may help to understand how they could affect the local transmission of circulating pathogens and how the risk of local transmission of native and imported pathogens is changed by invasive mosquito presence.

Mosquitoes with a recent blood meal in their abdomen could be used to trace their vertebrate host species. Different approaches have been used for the identification of mosquito’s blood meal sources, including precipitin test [77,78], gel diffusion [79,80] or enzyme-linked immunosorbent assay (ELISA) [81,82], and molecular techniques [83,84]. More recently, the matrix-assisted laser desorption ionization-time off light mass spectrometry (MALDI-TOF MS) has also been applied to mosquito’s blood meal identification. This is a proteomic technique based on the profiling of the blood meal proteins and the identification of the host by comparison with a reference database. This technique has already been used successfully for host identification in *Ae. albopictus* raised in laboratory, both for single and mixed blood meals [85,86], although there is still scarce evidence of its effectiveness identifying blood meals from field collected *Aedes* mosquitoes [87] given the large diversity of vertebrates present in wild communities. Another technique that has been recently used for blood-meal identification of mosquitoes is mid-infrared spectroscopy [88]. Although, to our knowledge, it has not been already used with invasive *Aedes* species, this methodology may represent an additional, less expensive and quicker alternative to other widely used methods [88]. Further information on the blood feeding patterns of these mosquito species could be obtained by exposing different hosts (e.g., humans or other animals) to mosquito attacks [89,90]. The novel mosquito electrocuting trap may allow researchers to identify the risk of exposure of humans to mosquito bites, including invasive *Aedes* species [91].

All of these different approaches for the identification of vertebrate hosts of mosquitoes have pros and cons that should be evaluated in terms of accessibility to specialized laboratory equipment, time, conservation of the samples, precision in host species identification and economic costs of analyses. For example, serological methods, such as ELISA or precipitin test, consist of the identification of hosts by exposing the blood to immunoglobulin G (IgG) conjugated against potential host species. Therefore, this technique is limited by the availability of antisera against some target species and the cross-reactivity between serum proteins in the case of closely related species limiting the range of hosts that can be identified [23,92]. Both of these techniques have been broadly used to identify blood meals of *Aedes* species [93,94,95]. To overcome the limitations regarding the availability of antisera and cross-reactivity, some of these studies used a combination of different techniques. For example, Savage et al. [96] used both precipitin test and ELISA to check for possible false positives, while Richards et al. [97] and Apperson et al. [81] employed PCR to conduct the specific identification of blood meals previously identified as avian-derived using ELISA. A similar procedure was used by Jansen et al. [92] with subsamples of blood meals that tested negative using an ELISA identification approach. On the other hand, the use of MALDI-TOF MS is still limited by the low number of species included in the database [85], although available information is progressively growing [98]. Molecular methods can be used to overcome this limitation, allowing increased specificity in the host identification. They consist of the amplification of sequences from different genes using either specific or universal primers. These techniques include DNA sequencing, use of group-specific primers, heteroduplex analysis, PCR-restriction fragment length polymorphism (PCR-RFLM), real-time PCR, reverse line-blot hybridation and DNA profiling [24]. DNA sequencing is the simplest and most specific method and is ideal for insects that feed on a wide range of vertebrate hosts or whose range of hosts is unknown. Once the sequence is obtained, matches can be found in available databases such as GenBank or the Barcode of Life Data System [24,99]. However, these approaches may be limited by factors including the gradual digestion of the blood meal that reduce the success of host identification [100,101] and the occurrence of partial blood meals that may not provide enough starting material [24]. In addition, DNA sequencing does also have constraints because it is time-consuming and relatively expensive, even more in the case of mixed-host blood meals. In addition, different sets of primers may match with sequences of the *Ae. albopictus* cytochrome (cyt) b gene, consequently amplifying the DNA of the mosquito (one unspecific extra locus) and not of the vertebrate hosts [102,103].

There are different genes that can be used as diagnostic markers for molecular blood meal identification, such as mitochondrial genes, ribosomal RNA genes, nuclear genes and repetitive DNA sequences, including micro and minisatellites [24]. Mitochondrial genes, especially cyt b and *c oxidase 1* (COI) genes, are, by far, the most broadly used genes for the molecular identification of mosquito blood meals sources. Within their advantages are the high number of copies and the high variability they present even between closely related species. However, nuclear genes have also been used successfully for identification of blood meals from vectors, but they present restrictions such as the low variability of the sequences in closely related species and that a low number of vertebrates have been characterized for these genes [104]. In addition, mammal blood cells are enucleated, limiting the use of these genes and highlighting the value of working with mitochondrial sequences [24].

## 4. Blood Feeding Patterns of Invasive *Aedes* Mosquitoes

We developed an extensive literature review on the blood feeding patterns of *Ae. aegypti*, *Ae*. *albopictus*, *Ae. japonicus* and *Ae. koreicus*—the four AIMs currently established in Europe. Studies considered here include those covering both their native and introduced distribution ranges. We used Google Scholar as the main search engine to find articles identifying vertebrate hosts of mosquitoes. We made searches with keywords including “Blood meal AND the scientific name of the mosquito species (e.g., *Aedes albopictus)”* (February, 2020) and “Feeding pattern AND the scientific name of the mosquito species” (March, 2020). This procedure was conducted for *Ae. albopictus, Ae. aegypti, Ae. japonicus* and *Ae. koreicus*. Additional references were obtained from the citations in these studies while other references were facilitated by colleagues. Overall, 276 studies were obtained at a first stage. We only selected studies based on the identification of blood meals of mosquitoes. From them, only those including information about the blood-feeding patterns of wild-caught invasive *Aedes* mosquitoes were selected. Studies that did not include data about the species focus of this study or that studied mosquitoes raised or fed in the laboratory were not used. Review articles were not considered here. As a result, we found 46 studies on the blood feeding patterns for four of the mosquito species that are included in this study. An Excel table was created including the information of interest, such as species studied, country where the study was developed, the habitat characteristics of the area of capture (i.e., urban, periurban or rural areas), total number of mosquitoes analyzed, methodology of blood meal identification, and the proportion of feedings obtained from different vertebrate hosts.

*Aedes albopictus* was the species most intensively studied with 31 articles focused on this species, followed by *Ae. aegypti*, *Ae. japonicus* and *Ae. koreicus,* which were included in 14, 8 and 2 studies, respectively. Overall, the dataset included in this study corresponded to 11,618 engorged mosquitoes. Of them, 6448 corresponded to *Ae. aegyti,* 4893 to *Ae. albopictus* and 227 to *Ae. japonicus*. Information of the vertebrate hosts from only 50 blood meals corresponded to *Ae. koreicus*. Most (80.4%; n = 37) of these studies were conducted in the invaded distribution range of these mosquito species, with only 11 of them being developed in Europe. Only five studies included data from both invaded and native distribution areas. No studies focusing on the identification of vertebrate hosts of *Ae. atropalpus* were found. Within these studies, 14 used serological methods, namely, ELISA, precipitin test or immunodiffusion technique, for the blood meal identification, while 27 used molecular methods and five combined both of them (four used ELISA and PCR and one used precipitin test and PCR).

**Figure 2:**
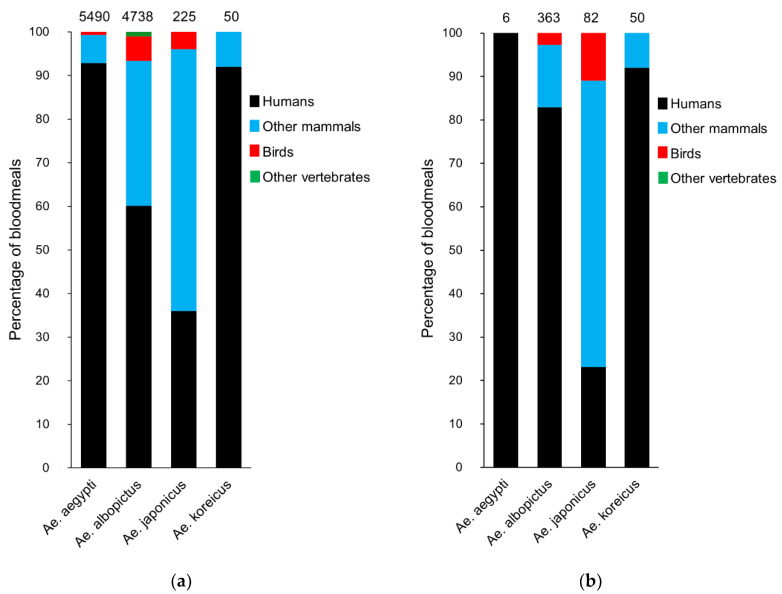
Percentage of blood meals derived from different vertebrate hosts. Percentage of feedings derived from humans (black), other mammals (blue), bird (red) and other vertebrates including reptiles, amphibians and fish (green) for the four *Aedes* invasive species studied (**a**) in both their native and invaded ranges or (**b**) studies conducted only in Europe. Blood meals derived from more than one vertebrate species (i.e., mixed blood meals) were excluded from the plot. The studies used for this figure are listed in the Supplementary Material Table S1.

According to the published information, 37 species were identified as vertebrate hosts of at least one of the AIM species studied, including 26 mammals and 11 birds. Ten other vertebrate groups were also identified as hosts of these AIMs, although the accuracy of identification reached levels above host genus. The broader host range was recorded for *Ae. albopictus* including 20 mammal and 5 bird species (Table 1), and *Ae. albopictus* was the only one that was documented to feed on ectotherms, including reptiles, amphibians and fish (Figure 2a). *Aedes aegypti* and *Ae. japonicus* fed on 5 mammal and 4 bird species and 15 mammal and 5 bird species, respectively. Only three species of mammals were identified as hosts of *Ae. koreicus*, with no records of birds. Results of the percentage of vertebrate host groups of the four AIM species are shown in Figure 2, including those studies developed in Europe (Figure 2b). Despite the ability of most of these species to feed on blood from different vertebrate groups, the vast majority of blood meals derived from mammals, representing over the 90% of the single blood meals identified. Interestingly, the anthropophilic behavior of these species is supported by the fact that humans represent 36–93% of the total single blood meals. Avian sources represented only 0–6% of the single blood meals.

The occurrence of mixed blood meals, those containing blood from two or more vertebrate host species, was highly variable between studies. The proportion of these mixed meals ranged from <1% to 78% of the total feedings for *Ae. aegypti*, and from 3% to 100% for *Ae. albopictus,* although this extremely high value was reported by a study where only seven individuals were analyzed [82]. For the case of *Ae. japonicus*, authors recorded a percentage of 8% and 50% of mixed blood meals in the only two studies recording its occurrence, although the highest value was obtained in a study including only two mosquitoes [105]. To our knowledge, the occurrence of mixed blood meals has not been reported for *Ae. koreicus*, likely due to the low number of studies and mosquitoes analyzed for this species.

## 5. Concluding Remarks and Future Prospects

Some of the AIMs studied, particularly *Ae. albopictus* and *Ae. japonicus*, have a wide range of hosts, feeding on a broad diversity of vertebrates. All the AIMs had a marked feeding preference for mammals, especially to feed on humans. In particular, human blood represented over 90% of the blood meals identified for *Ae. aegypti* and *Ae. koreicus* (Figure 2). This large percentage of human-derived blood meals could be explained, at least in part, due to the nature of the collection sites. Although sampling sites include urban, periurban and rural areas [93,129,130], where the availability of humans may differ, most studies have been conducted in urban environments, especially when studying *Ae. albopictus* and *Ae. aegypti*. *Aedes aegypti* has a highly anthropophilic behavior, entering houses to feed on human blood and breeding in man-made containers in most of its populations [131]. Birds, and other vertebrates, have been also recorded as potential hosts for these AIMs, although they represent a low percentage of the mosquito blood meals studied. *Aedes albopictus* was the species with the highest percentage of avian blood meals, and the only one found to feed on ectotherms. When focusing on studies developed in Europe, a slight change can be appreciated in the proportions of the blood meal sources in *Ae. albopictus* and *Ae. japonicus. Aedes albopictus* shows an increase in the proportion of feedings obtained from humans, which is not surprising given the fact that all six studies had collection sites in urban environments, and only two combined urban and periurban environments. *Aedes japonicus*, on the contrary, presents a decrease in the anthropophilic pattern. Again, this can be caused by the nature of the collection sites, since the mosquitoes were captured in a zoo and in two resellers of used tires. Nevertheless, the data collected in Europe are scarce, including the study of a relatively low sample size of mosquitoes collected in a handful of countries. Thus, it would be desirable to develop additional studies in Europe to understand seasonal and spatial variation in AIM blood feeding behavior and the impact of urbanization on mosquito feeding patterns and its potential consequences for pathogen transmission in villages and cities. Based on these results and information of their vector competence, AIMs may play an important role in the transmission of pathogens circulating between mammals, especially humans. However, although avian pathogens such as avian malaria have been detected in *Ae. albopictus*, its relevance may be considered low compared to other species such as *Culex pipiens* [84,103,106]. This may be also the case for the transmission of zoonotic pathogens of avian origin such as West Nile virus, in spite that this species has been reported as a potential bridge vector between birds and humans in invaded ecosystems [132].

Interestingly, studies on the feeding preferences of mosquitoes are still scarce as most of the published information did not consider the abundance of the potential vertebrate hosts in the studied localities. This could lead, for example, to an overestimation of the anthropophilic behavior of AIMs in studies developed exclusively in urbanized environments where humans may represent the most common available hosts. In fact, a recent study identified urbanization as a key component explaining the variation of *Ae. aegypti* preference for human odors [133]. This anthropophilic behavior showed in urbanized areas [103,107] could facilitate the transmission of mosquito-borne pathogens and facilitate local transmission arising from virus imported by infected travelers. Thus, future studies on the feeding pattern of AIMs may be carried out combined with censuses of vertebrate hosts in the area.

This review also highlights other limitations of the current knowledge of the feeding patterns of AIMs in Europe due to the extremely low number of mosquitoes analyzed for some species. This is particularly the case for *Ae. koreicus*, for which records of blood meal hosts have been obtained for only 50 mosquitoes. These numbers are even lower when considering just the blood meals analyzed from European populations of mosquitoes. It is important to highlight that studies focused on AIMs are still scarce in Europe. Specifically, we found only ten studies that analyzed the feeding patterns of AIMs in the area, but they were developed in areas corresponding to only five countries (Spain, Italy, the Netherlands, Switzerland and Madeira (Portugal). This fact may be partially due to the limited distribution range of most of these mosquito species in Europe. However, the blood feeding patterns of *Ae*. *albopictus* have only been studied in Spain, Italy and Switzerland [73,84,103,106,112,129], despite being currently established in more than 15 European countries [31]. In addition, previous studies have not analyzed the factors affecting spatial or seasonal variation in blood meal composition or in the incidence of human blood meals in this species. Furthermore, we found no data on the feeding patterns of *Ae. atropalpus.* This is not surprising in the case of Europe, since the species was eradicated from different countries and nowadays there are not known established populations, although it is present in a small area in the Netherlands [35].

Overall, these findings highlight a major gap of knowledge that should be fulfilled in the future to finally understand how the establishment of *Aedes* species has changed the patterns of transmission of mosquito-borne pathogens in Europe and the risk of local outbreaks of imported arboviruses and other imported pathogens.

In addition, molecular and serological techniques can be used to identify pathogens present in the abdomen of blood-engorged insects (e.g., xenosurveillance) [134,135]. This procedure allows researchers to obtain information not only about mosquitoes feeding preferences, but also which pathogens are interacting with them and are circulating in the populations.

## Figures and Tables

**Table 1:** Vertebrate hosts of the four invasive *Aedes* species identified using molecular methods.

*Aedes albopictus*	Family	Species	References
Mammals	*Hominidae*	*Homo sapiens*	[73,84,97,103,105,106], [107] *, [108,109,110,111,112,113], [114] *, [115], [116] *, [117]
	*Canidae*	*Canis lupus* **	[97,108,109,110,113], [114] *, [115,117]
	*Felidae*	*Felis silvestris* **	[97,105,106], [107] *, [109], [114] *, [117]
	*Procyonidae*	*Procyon lotor*	[97], [116] *
	*Muridae*	*Rattus norvegicus*	[110,113], [116] *, [117]
		*Mus musculus*	[105]
	*Cricetidae*	*Peromyscus leucopus*	[109]
	*Sciuridae*	*Sciurus carolinensis*	[111]
	*Leporidae*	*Sylvilagus floridanus*	[109], [116] *
	*Suidae*	*Sus* **	[108,111], [114] *, [115]
	*Bovidae*	*Bos taurus*	[73]
	*Cervidae*	*Odocoileus virginianus*	[117]
	*Equidae*	*Equus caballus*	[97]
	*Soricidae*	*Suncus murinus*	[111]
	*Erinaceidae*	*Erinaceus europaeus*	[84]
	*Dasypopidae*	*Dasypus novemcintus*	[116] *
	*Phyllostomidae*	*Tonatia bidens*	[110]
	*Didelphidae*	*Didelphis virginiana*	[97,109]
Birds			
	*Phasianidae*	*Gallus domesticus*	[97,115]
	*Turdidae*	*Turdus merula*	[84]
	*Passeridae*	*Passer montanus*	[84]
	*Tamnophilide*	*Taraba major*	[110]
	*Cardinalidae*	*Cardinalis cardinalis*	[97]
	*Anatidae*	Unknown	[115]
*Aedes aegypti*			
Mammals	*Hominidae*	*Homo sapiens*	[92,116,118,119,120,121]
	*Canidae*	*Canis lupus* **	[92,116,118,120]
	*Felidae*	*Felis silvestris* **	[92,120]
	*Bovidae*	*Bos taurus*	[92,118]
	*Suidae*	*Sus scrofa* **	[118]
	*Equidae*	*Equus caballus*	[120]
Birds			
	*Phasianidae*	*Gallus domesticus*	[120]
	*Phasianidae*	*Francolinus squamatus*	[119]
	*Mimidae*	*Mimus polyglottos*	[116]
	*Musophagidae*	*Crinifer piscator*	[119]
*Aedes japonicus*			
Mammals	*Hominidae*	*Homo sapiens*	[81,117,122,123,124,125]
	*Canidae*	*Canis lupus* **	[122]
		*Canis latrans*	[126]
	*Felidae*	*Felis silvestris* **	[117]
		*Panthera leo persica*	[122]
	*Procyonidae*	*Procyon lotor*	[126]
	*Phocidae*	*Phoca vitulina*	[122]
	*Muridae*	*Rattus norvegicus*	[117]
	*Sciuridae*	Unknown species	[123]
	*Camelidae*	*Lama* sp.	[122]
	*Bovidae*	*Bos taurus*	[125]
		*Boselaphus tragocamelus*	[122]
		*Ovis* sp.	[122]
	*Cervidae*	*Dama dama*	[124]
		*Odocoileus virginianus*	[123,124,126]
	*Equidae*	*Equus caballus*	[81,124]
		*Equus asinus*	[122]
	*Didelphidae*	*Didelphis virginiana*	[124]
Birds			
	*Phasianidae*	*Gallus domesticus*	[122]
	*Turdidae*	*Turdus merula*	[122]
	*Passeridae*	*Passer domesticus*	[122]
	*Spheniscidae*	*Spheniscus humboldti*	[122]
	*Rheidae*	*Rhea pennata*	[122]
*Aedes koreicus*			
Mammals	*Hominidae*	*Homo sapiens*	[127,128]
	*Canidae*	*Canis lupus* **	[127,128]
	*Bovidae*	*Bos taurus*	[128]

* These also reported blood meal hosts such as “amphibian”, “fish” or “turtle”. ** The identification of blood from *Canis lupus*, *Sus scrofa* and *Felis silvestris* may correspond to domestic animals.

## Supplementary Materials

The following are available online at https://www.mdpi.com/2075-4450/11/12/848/s1, Table S1: Vertebrate hosts of *Aedes* invasive species identified using different approaches.
